# Supplementary material for: Whole-Transcriptome Sequencing Reveals a ceRNA Regulatory Network Associated with the Process of Periodic Albinism under Low Temperature in Baiye No. 1 (Camellia sinensis)
Source: Int J Mol Sci. 2023 Apr 12;24(8):7162. doi: 10.3390/ijms24087162 (PMC10138444; doi:10.3390/ijms24087162)
Supplement: Supplementary file 1 [file ijms-24-07162-s001.zip › Table S1 Overview of the RNA-Seq Datas and miRNA-Seq Datas.pdf]

Table S1.1. Overview of the RNA-Seq Datas and reference genome mapped results.

Samples	Raw reads	RawData(bp)	Clean reads	CleanData(bp)	Q20(%)	Q30(%)	GC(%)	Removed rRNA	Mapped reads(%)	Unique_Mapped(%)	Multiple_Mapped(%)
Bud-1	121569626	18.24G	121102268 (99.62%)	17.64G (96.71%)	96.47	91.05	46.60	113404264 (93.64%)	76722498 (67.65%)	47325338 (63.31%)	11434042 (15.30%)
Bud-2	109279262	16.39G	108909446 (99.66%)	15.97G (97.44%)	96.28	90.42	43.16	104974920 (96.39%)	74158539 (70.64%)	64251800 (61.07%)	15698074 (14.92%)
Bud-3	109963048	16.49G	109581908 (99.65%)	16.08G (97.51%)	96.43	90.77	42.74	105136034 (95.94%)	76398040 (72.67%)	73480089 (62.92%)	18133746 (15.53%)
Alb-1	77434510	11.62G	77208396 (99.71%)	11.35G (97.68%)	96.54	91.11	43.90	74753050 (96.82%)	58759380 (78.60%)	66163432 (58.34%)	10559066 (9.31%)
Alb-2	109010414	16.35G	108513338 (99.54%)	16.00G (97.86%)	96.23	90.56	43.55	105215746 (96.96%)	79949874 (75.99%)	63769773 (60.75%)	10388766 (9.90%)
Alb-3	120095866	18.01G	119735168 (99.70%)	17.68G (98.17%)	96.45	90.95	43.58	116787752 (97.54%)	91613835 (78.44%)	66050997 (62.82%)	10347043 (9.84%)
Med-1	94374862	14.16G	94198536 (99.81%)	13.83G (97.67%)	97.08	91.81	43.62	142140754 (98.85%)	122408728 (98.96%)	75180138 (61.42%)	26117472 (21.34%)
Med-2	85076616	12.76G	84843658 (99.73%)	12.62G (98.90%)	97.33	92.58	44.21	78841794 (92.93%)	121627678 (98.40%)	77025801 (63.33%)	22220870 (18.27%)
Med-3	86468662	12.97G	86257148 (99.76%)	12.80G (98.69%)	97.53	93.00	44.20	84143848 (97.55%)	106730618 (98.65%)	64612619 (60.54%)	22873858 (21.43%)
Gre-1	123991834	18.60G	123692874 (99.76%)	18.37G (98.76%)	96.59	91.06	43.63	122408728 (98.96%)	101297610 (82.75%)	59684968 (63.69%)	16118555 (17.20%)
Gre-2	124037732	18.61G	123606000 (99.65%)	18.33G (98.50%)	96.76	91.63	43.85	121627678 (98.40%)	99246671 (81.60%)	48132295 (61.05%)	17381559 (22.05%)
Gre-3	108555700	16.28G	108186182 (99.66%)	16.08G (98.77%)	96.47	91.01	43.71	106730618 (98.65%)	87486477 (81.97%)	52424495 (62.30%)	16304937 (19.38%)
total	1269858132	190.48G	1265834922(99.68%)	186.75G (98.04%)	96.68	91.33	43.90	1227738338 (96.68%)	955679733 (75.26%)	758101745 (62.30%)	197577988 (62.30%)

Table S1.2. Summary of miRNA sequencing data.

Samples	Clean reads	High quality	3'adapter null	Insert null	5'adaptercontaminants	smaller_than_18nt	polyA	clean_tags
Bud1	15806283	15689358 (99.2603%)	21672 (0.1381%)	68669 (0.4377%)	39275 (0.2503%)	1116726 (7.1177%)	2384 (0.0152%)	14440632 (92.0409%)
Bud2	13616643	13511738 (99.2296%)	20596 (0.1524%)	59263 (0.4386%)	141095 (1.0442%)	2518141 (18.6367%)	699 (0.0052%)	10771944 (79.7229%)
Bud3	13353403	13232309 (99.0932%)	25785 (0.1949%)	67023 (0.5065%)	145721 (1.1013%)	3161004 (23.8885%)	420 (0.0032%)	9832356 (74.3057%)
Alb1	13228633	13125013 (99.2167%)	21299 (0.1623%)	73839 (0.5626%)	135440 (1.0319%)	3169307 (24.1471%)	528 (0.0040%)	9724600 (74.0921%)
Alb2	12949510	12867899 (99.3698%)	8766 (0.0681%)	61816 (0.4804%)	119012 (0.9249%)	2869986 (22.3035%)	435 (0.0034%)	9807884 (76.2198%)
Alb3	12482122	12405098 (99.3829%)	17461 (0.1408%)	56622 (0.4564%)	214940 (1.7327%)	3538337 (28.5232%)	239 (0.0019%)	8577499 (69.1450%)
Med1	12172855	12064952 (99.1136%)	9584 (0.0794%)	68027 (0.5638%)	144320 (1.1962%)	2630502 (21.8028%)	233 (0.0019%)	9212286 (76.3558%)
Med2	15382397	15284279 (99.3621%)	15368 (0.1005%)	77174 (0.5049%)	226839 (1.4841%)	3926941 (25.6927%)	307 (0.0020%)	11037650 (72.2157%)
Med3	12159100	12089671 (99.4290%)	13008 (0.1076%)	60765 (0.5026%)	59337 (0.4908%)	1230106 (10.1749%)	408 (0.0034%)	10726047 (88.7208%)
Gre1	14677490	14540835 (99.0689%)	14537 (0.1000%)	71897 (0.4944%)	161883 (1.1133%)	3452523 (23.7436%)	330 (0.0023%)	10839665 (74.5464%)
Gre2	12745933	12637810 (99.1517%)	11770 (0.0931%)	57884 (0.4580%)	174943 (1.3843%)	3338089 (26.4135%)	182 (0.0014%)	9054942 (71.6496%)
Gre3	16468412	16307117 (99.0206%)	16350 (0.1003%)	69481 (0.4261%)	35842 (0.2198%)	1237032 (7.5858%)	1411 (0.0087%)	1494700 1(91.6594%)
total	165042781	163756079 (99.2204%)	196196 (0.1189%)	792460 (0.1076%)	1598647 (0.9686%)	32188694 (19.5032%)	7576 (0.0046%)	128972506 (78.1449%)
